# A reliability analysis: human trafficking curriculum assessment tool (HT-CAT) for health care provider human trafficking trainings

**DOI:** 10.1186/s12909-025-06932-2

**Published:** 2025-03-21

**Authors:** Hanni Stoklosa, Xichen Wang, Boci Meng, Jason Rydberg, Adebimpe Adewusi, Madison Murphy Barney, Kendra Glassman, Kara Napolitano, Claudia Wald, Lela Bachrach

**Affiliations:** 1 Department of Emergency Medicine, Brigham and Women’s Hospital Harvard Medical School, Boston, MA USA; 2 HEAL Trafficking, Long Beach, CA USA; 3 School of Criminology and Justice Studies, University of Massachusetts Lowell, Lowell, MA USA; 4 School of Criminology and Justice Studies, Center for Program Evaluation, University of Massachusetts Lowell, Lowell, MA USA; 5 CARES Northwest, Randall Children’s Hospital, Legacy Emmanuel Medical Center, Portland, OR USA; 6 Harvard T.H. Chan School of Public Health, Middlesex, VT USA; 7 Denver Health, Firestone, CO USA; 8 Laboratory to Combat Human Trafficking, Denver, CO USA; 9 New York, NY USA; 10 Department of Pediatrics, University of California San Francisco, San Francisco, CA USA

**Keywords:** Human trafficking, Health professions education, Labor trafficking, Sex trafficking, Healthcare training, Curriculum assessment, Trauma-informed care, Lived-experience, Medication education, Healthcare policy

## Abstract

**Background:**

Human trafficking is an egregious human rights violation with profound physical and mental health sequelae. Healthcare encounters present critical opportunities to identify and support individuals experiencing trafficking. To maximize these opportunities, healthcare professionals must be equipped to recognize the signs of trafficking and respond effectively with trauma-informed, survivor-centered care. High quality training is essential to ensure that healthcare providers have the knowledge and skills to fulfill this role. To address the need for standardized, evidence-based evaluation of trafficking training, the Human Trafficking Curriculum Assessment Tool (HT-CAT) was developed as a structured quality assessment tool. This study evaluates HT-CAT’s reliability.

**Methods:**

The Human Trafficking Curriculum Assessment Tool (HT-CAT) was used to evaluate the quality of a compendium of introductory human trafficking educational resources for health care professionals. Twenty-four trainings were systematically reviewed using the HT-CAT by a minimum of three reviewers. Statistical analyses were conducted to examine interrater reliability and to describe variation across trainings and assessment tool items.

**Results:**

There was high interrater agreement based on summed HT-CAT domain scores (ICC = 0.88, 95% CI 0.84–0.93). Individual item scores had variable agreement (K = 0.30, 95% CI 0.22–0.38) with high between training variability, as well as variable agreement on how specific items should be scored (alpha = 0.27, 95% CI 0.23–0.31). This variation in scoring was consistent between items, suggesting that there were no items that systematically produced more or less agreement than others.

**Conclusions:**

The HT-CAT is a reliable tool for assessing the overall quality of human trafficking education of health professionals. HT-CAT has important applications for health policy makers and health professions educators. Rigorous assessment of key components of human trafficking trainings for healthcare professionals can help ensure that high quality, relevant education is offered to this group of direct service providers that play an important role in supporting survivors of human trafficking. High-quality training equips providers, a vital lifeline for survivors of human trafficking, with the knowledge and tools to offer compassionate, informed care.

**Supplementary Information:**

The online version contains supplementary material available at 10.1186/s12909-025-06932-2.

## Background

### Introduction

Human trafficking is an egregious human rights violation which involves traffickers profiting from the exploitation of others for labor and/or sex [1]. It is a multi-billion dollar criminal industry that impacts millions of people globally [2]. Trafficking disproportionately impacts marginalized communities. According to the International Labour Organization, an estimated 50 million people are impacted by extreme exploitation worldwide [3]. Data from the United States National Human Trafficking Hotline identified 16,658 potential victims of trafficking in 2020 alone [4]. This is an underestimate of the true prevalence given that these numbers reflect only those victims who sought resources via a national hotline. The overwhelming majority of human trafficking cases go unreported each year [5]. 

Health care professionals are uniquely positioned to recognize trafficking, treat health complications, and refer patients to appropriate support resources. Patients experiencing trafficking may present to health care settings with any of the myriad physical and mental health sequelae of trafficking situations [6–8]. In fact, studies have shown that between 28 and 88% of people being trafficked in the United States report being cared for in a healthcare setting during their period of exploitation [9–11]. It is not only imperative for clinicians to be aware of trafficking but to respond with compassion and helpful linkages to relevant community based organizations and agencies. Many victims of trafficking are afraid or mistrustful of health professionals and health systems. Therefore, beyond recognizing the issue and identifying the indicators of trafficking among patients, a trauma-informed response is equally important [12, 13].

Quality training on human trafficking is vitally important for healthcare professionals. As awareness of human trafficking as a public health issue grows, so does the demand for effective training among health care providers. Several states now require training to help providers recognize and respond to human trafficking [14]. Health professionals need access to high-quality, accessible, and comprehensive training programs that are evidence-based, trauma-informed, and culturally responsive. The recently published core competencies for human trafficking response in health care and behavioral health systems emphasize the importance of these principles [15]. 

A decade ago, a review highlighted the urgent to develop, implement, and evaluate high-quality education and training programs on human trafficking for healthcare providers [16, 17]. To address this need, HEAL Trafficking and the Laboratory to Combat Human Trafficking developed the Human Trafficking Curriculum Assessment Tool (HT-CAT), a structured evaluation framework designed to assess introductory human trafficking trainings across key domains (Table 1). HT-CAT systematically identifies strengths and gaps within a training, offering actionable insights to enhance content quality, ensure trauma-informed approaches, and align with best practices in healthcare education [18]. 

Since its development, HT-CAT has gained significant recognition. It was featured in the 2020 U.S. State Department Trafficking in Persons Report and has been adopted as a standard-setting metric in human trafficking curriculum development across eight countries. In the United States, the state of Texas utilizes HT-CAT to implement its health professional human trafficking education mandate [19]. 

The tool is designed as a three-page checklist comprising 34 key assessment questions, along with an additional page of citations and resources to support curriculum development and refinement. By providing a structured and systematic approach to evaluating human trafficking training, HT-CAT helps ensure that health professionals receive the education needed to effectively identify and support trafficking survivors.

The purpose of the current research is to consider the practical application of HT-CAT, using reliability metrics to examine the extent to which knowledgeable raters reviewing the same curriculum would arrive at similar conclusions regarding its features [20].

## Methods

### Setting

HEAL Trafficking, the international health care nonprofit focused on trafficking as a public health issue, created a compendium of available human trafficking training resources for health care professionals between 2015 and 2019. The compendium was based on multiple literature reviews [18], as well as repeated open calls to the HEAL network for members to add relevant trainings. The trainings were all web-based media such as online modules and webinars rather than literature such as review articles or other educational material. This led to a compendium of 58 English-language trainings meant to inform, educate, or offer guidance to healthcare professionals on human trafficking.

**Table 1:** Human trafficking curriculum assessment tool (HT-CAT) domains

Domain	Aggregate Score (Points Possible)	Sample items
Overview and Definitions	9	Definition of trafficking, vulnerabilities to trafficking, TVPA, trafficking vs. smuggling, etc.
Health Impact	4	Injuries, chronic medical problems, mental health sequelae, sexual health, quality of life impacts, etc.
Identification & Assessment	11	Indicators of trafficking, barriers to disclosure, trauma-informed care, safety, use of professional interpreters, etc.
Response & Follow Up	8	How to intervene, mandatory reporting, leveraging community resources, etc.
Design	2	Survivor consultation, exclusion of sensationalized imagery

### Assessment

HT-CAT was designed to evaluate a basic 101 level of human trafficking trainings for health professionals, so exclusion criteria were applied to the compendium. From the initial frame of 58 trainings, exclusion criteria resulted in the elimination of trainings based on their specialized nature (for example, focusing more on domestic violence, sexual assault response, trauma, etc.), educational resources primarily for non-health audiences, technical issues (such as defunct links), financial barriers to access, and for redundancy. This yielded 24 human trafficking trainings for analysis. The researchers created a version of HT-CAT in Qualtrics with key domains (Table 1) and items (Additional File A) to match HT-CAT. The HT-CAT domains include “Design,” “Overview,” “Health Impact,” “Identification & Assessment,” and “Response & Followup.” After familiarizing themselves with HT-CAT, each of these 24 trainings was reviewed by three or more reviewers, with an average of four reviews (range 3–6 reviews). The review taskforce occurred between June 2019 and December 2019. The results of these reviews were exported from Qualtrics into a spreadsheet for analysis. The individual trainings are presented in a de-identified fashion using arbitrary identifiers (e.g., “Training 1”). The list of trainings reviewed can be found in the Additional File B.

### Statistical analysis

The overarching purpose of this analysis was to examine interrater reliability to determine the extent to which different individuals would extract similar information when reviewing the same training curricula, with a focus on describing variation across different trainings (i.e., did raters on a specific training agree, and did this vary across trainings) as well as specific items within each training (i.e., on which items were raters most/least likely to agree). A variety of statistical tools for assessing interrater reliability were employed, each of which is informed by the level of measurement for the quantities being assessed and are similar to those employed in comparable efforts [21, 22]. 

For each training, the intraclass correlation (ICC) was used to assess agreement between different raters on the basis of summed domain scores. This metric is commonly used to assess agreement between multiple coders for numeric variables, where values in excess of 0.75 are considered an indicator of excellent agreement [23]. The ICC was calculated using the function icc from the R package irr, using a two-way model [24]. To consider between training interrater reliability on the basis of scoring for individual assessment items we used Fleiss’ kappa via irr::kappam.fleiss [22]. This statistic is applicable given that each individual item represents a binary score, and there are multiple raters used for each training. Kappa values in excess of 0.4, 0.6, and 0.8 suggest moderate, substantial, and almost perfect agreement, respectively [25]. 

In order to assess reliability on specific items (i.e., across all trainings, for a specific item and for any given set of *n* raters, to what extent was there agreement on how the specific item should be scored), we split the data into 34 item specific matrices, where each row represented a specific training, and the scoring for the designated raters was spread across columns. We used Krippendorf’s alpha to examine variation in agreement between specific items, calculated via the R package krippendorfsalpha [26]. This IRR statistic is useful given that the number of raters varies from training to training, where values in excess of 0.8 suggest satisfactory agreement [27]. 

For each analysis, we present relevant IRR statistics and uncertainties for each training, or for each assessment item. These quantities are then pooled using a Dersimonian-Laird random effects meta-analysis in order to obtain an overall estimate of reliability. This analysis was executed using the function rma with method = “DL” in the R package metafor [28]. Practically, to conduct the DerSimonian-Laird random effects model, both the effect size and the variance of the effect size were required for each reliability statistic. For Fleiss’ Kappa and the ICC, the variance was estimated via the bootstrap, a resampling method that involves repeatedly sampling with replacement from the data and re-estimating the reliability metric on each replicate to estimate the standard error. This procedure was performed using the R package boot (Canty and Ripley, 2024) [29]. The use of bootstrapped standard errors was necessary primarily because the function irr::kappam.fleiss, which estimates Fleiss’ Kappa, does not provide an analytic standard error. Therefore, the bootstrap offers a robust mechanism for computing uncertainties regardless of the underlying distribution [30]. 

For the DerSimonian-Laird random effects model applied to Krippendorff’s alpha, the analytic standard error provided by the R package krippendorfsalpha was used. This package also offers confidence intervals, which were converted into standard errors for the analysis. Although consistency in variance estimation across the different reliability metrics would be desirable, the data configuration used to produce the Krippendorf’s alpha (i.e., an item-specific dataset where every row has ratings for a specific item from a specific training) had an unequal number of observations across columns due to variation in the number of raters. This configuration produces missing observations, making bootstrapping infeasible for estimating standard error in this context. Instead, the analytic standard errors provided by the package were used to conduct the DerSimonian-Laird random effects model for Krippendorff’s alpha.

## Results

### Summed domain scores

**Fig. 1:**
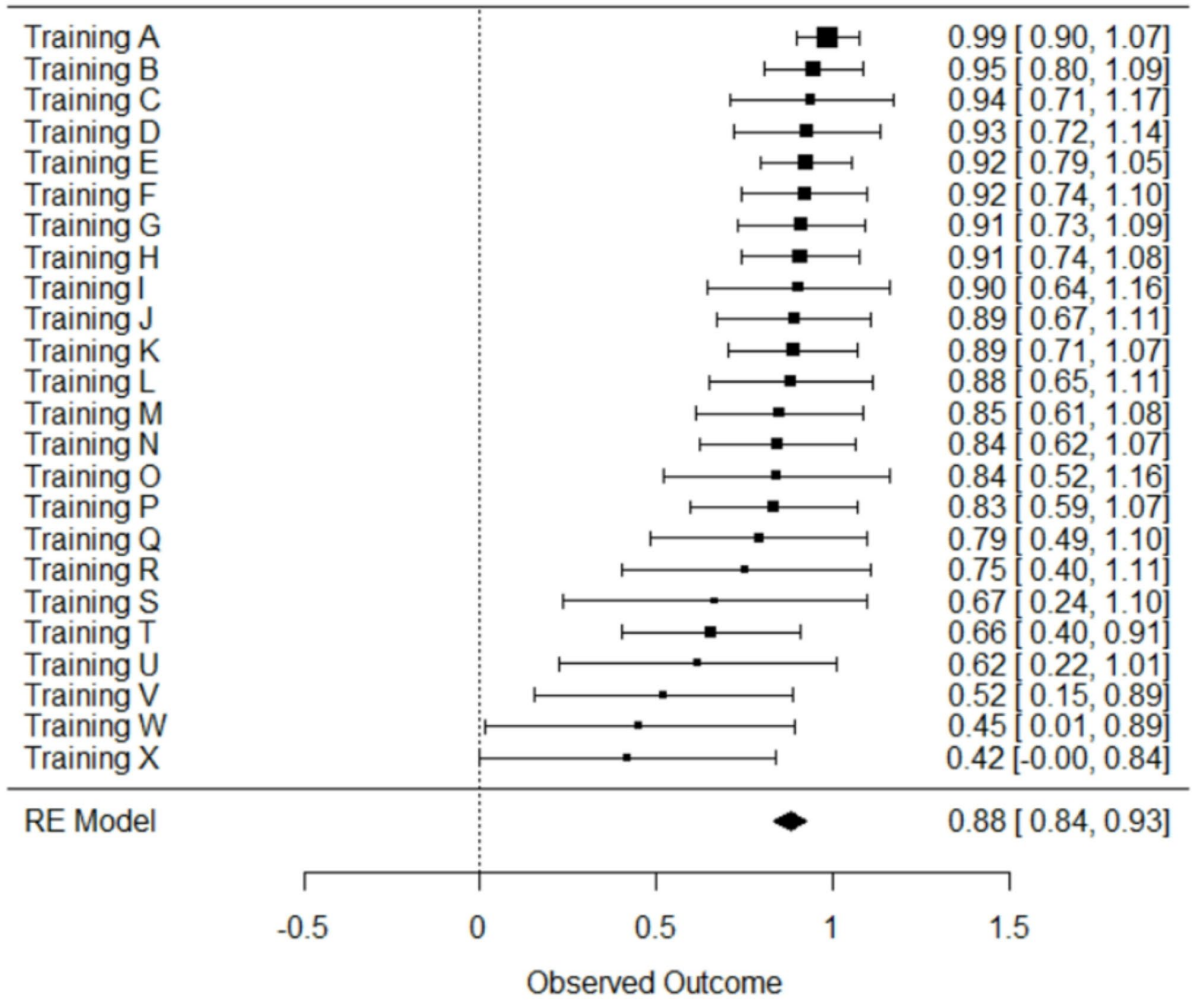
Forest plot for interrater reliability on summed domain scores - ICC and 95% confidence interval

Our initial assessment of interrater reliability considers whether different raters arrive at similar summed domain scores when scoring the same training curriculum. For instance, considering the HT-CAT domains and scores displayed in Table 1, for any given training to what extent did the different raters arrive at similar scores for each domain? The results of this assessment of interrater reliability is displayed in Fig. 1, where each ICC quantifies the extent of agreement among raters assigned to each of the individual trainings. The overall ICC estimate derived from the random effects meta-analysis was 0.88 (95% CI of 0.84–0.93), suggesting good to excellent interrater reliability (Koo & Li, 2016). There was also relatively little heterogeneity between trainings (I^2 = 22.6%, Q_23 = 29.7, *p* =.157), suggesting that based on summed domain scores there was little systematic variation in certain trainings producing more or less rater agreement than others.

### Individual item scoring

**Fig. 2:**
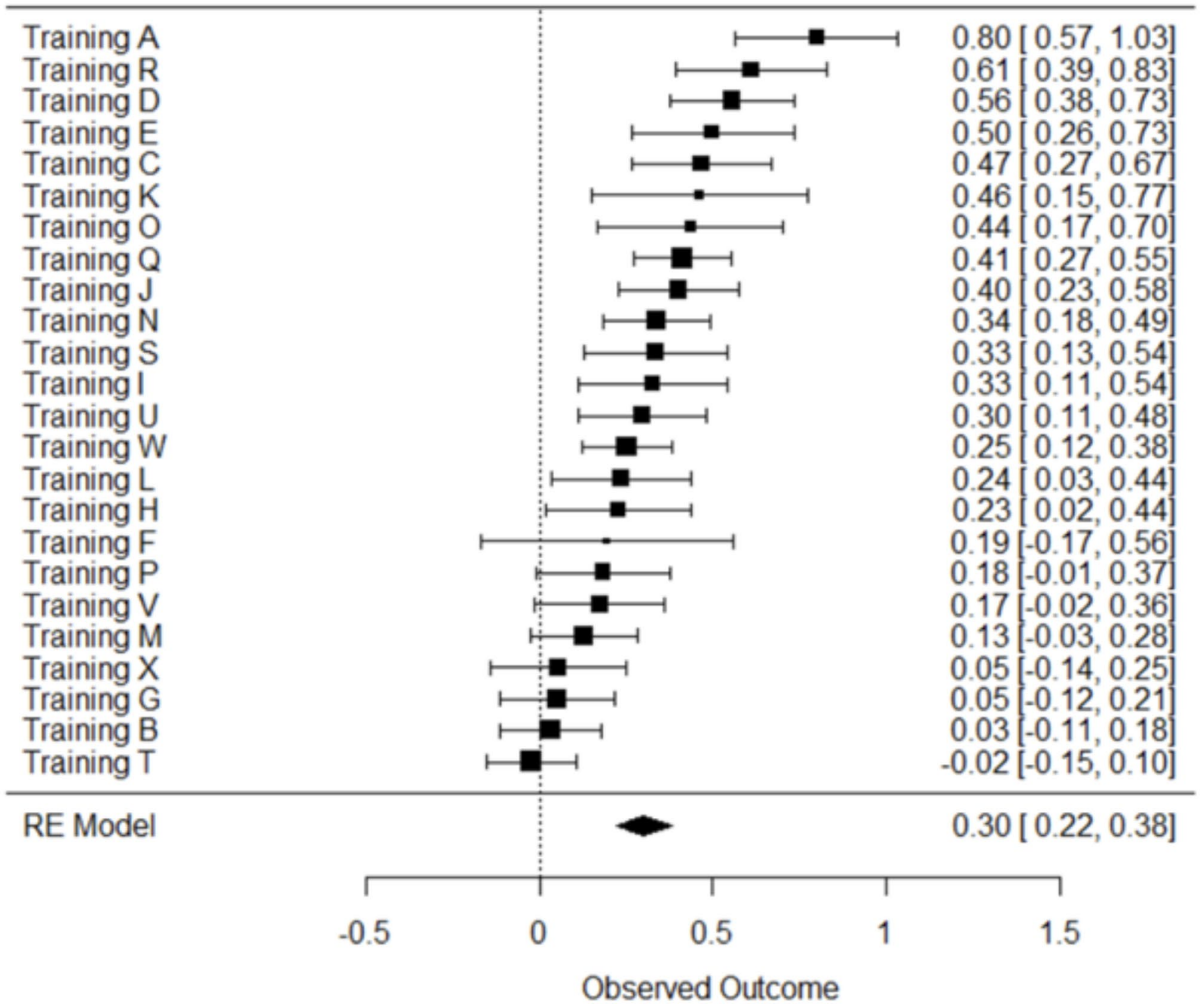
Forest plot for interrater reliability based on individual item agreement - Fleiss’ Kappa and 95% confidence interval

Following the assessment of interrater reliability based on summed domain (Table 1) scores, we then examined the extent of agreement based on how raters scored each individual HT-CAT item. For instance, for any given training, to what extent did the assigned raters agree on how each individual item should be scored? Fig. 2 displays the assessment of reliability based on rater agreement on individual HT-CAT items. The meta-analytic estimate for Fleiss’ Kappa (K = 0.30, 95% CI 0.22–0.38) suggests relatively poor agreement between raters on any given training. Compared to the summed domain scores, there was considerably more variability among training heterogeneity (I^2 = 78.7%, Q_23 = 107.7, *p* = < 0.001), suggesting that variation within domains was relatively higher than variation between domains.

### Between item variation

**Fig. 3:**
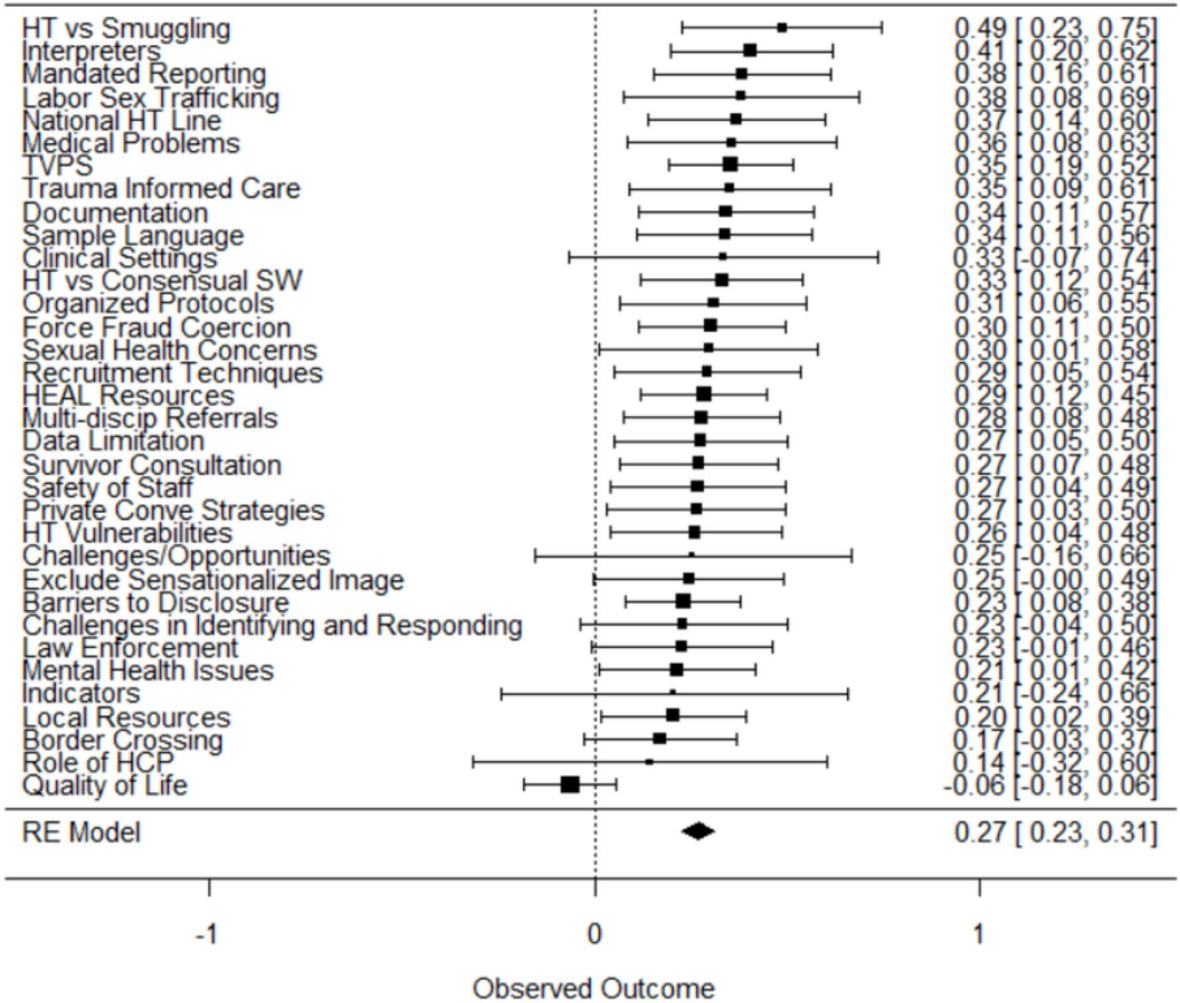
Forest plot for interrater reliability based on between item variation - Krippendorf’s alpha and 95% confidence interval

The final analysis component considered whether there were any specific items on the HT-CAT that produced systematically higher or lower agreement than others. For instance, imagine creating a dataset comprised of all the rater scores for a specific item (e.g., “Are all major forms of human trafficking discussed?” or “Does the training exclude sensationalized imagery?”), and comparing agreement among the raters for that item across all trainings - how much of the variation in agreement occurs among the raters, and how much is between the different items? Fig. 3 presents the assessment of interrater agreement based on how assigned raters for any given training agreed on the scoring for the 34 specific items in the HT-CAT. The overall Krippendorf’s alpha from the random effects meta analysis was 0.27 [95% CI 0.23–0.31], suggesting relatively poor agreement between raters on specific items. Meta-analytic metrics indicated that there was relatively little variation between items (I^2 = 20.5%; Q_33 = 41.5, *p* =.148), suggesting that it is difficult to contend that specific items produce more or less agreement than others. Instead, the majority of the variation in scoring occurs among raters scoring within any given item.

## Discussion

The results of our analysis show that HT-CAT is a reliable tool for assessing introductory-level human trafficking trainings for healthcare professionals. As such, HT-CAT can be helpful for those overseeing human trafficking education implementation in health systems, clinician training schools, for credentialing bodies as well as policy makers. Although reviewers’ scores varied for individual items, their overall scores for the trainings and within each domain were similar. Since healthcare providers are essential in recognizing people experiencing human trafficking and supporting affected individuals, it is crucial to provide them with high-quality, evidence-based education and training. Moreover, health care trainings which share misinformation or provide a non-comprehensive snapshot of trafficking situations has the potential to result in harm for people experiencing trafficking who access healthcare [31]. 

Since the launch of this analysis, efforts to outline core competencies for health professionals for trafficking have been published by the U.S. Department of Health and Human Services, making our tool even more essential for guiding implementation and ensuring that professionals effectively integrate these competencies into their practice [32]. Furthermore, each year more states are mandating education on interpersonal violence, including trafficking as part of their recredentialing processes [33]. Having reliable standards for measurement of fulfillment of these competencies is vital.

The ultimate goal of any clinical training is to improve the lives of patients [19]. While our study highlights the reliability of HT-CAT in assessing training quality, future research should investigate how training translates into clinical practice and ultimately impacts patient care. Specifically, studies should assess how human trafficking training influences healthcare professionals’ knowledge, attitudes, and practices, as well as the extent to which these changes lead to improved health outcomes for trafficking survivors [17, 34, 35]. Understanding how trained clinicians identify, engage with, and provide trauma-informed care to trafficking survivors is crucial [36]. Additionally, research should explore whether enhanced provider training reduces barriers to care, improves survivor trust in healthcare, and facilitates appropriate referrals to support services [37]. 

Given the complexity of human trafficking and its intersection with social determinants of health, future studies should also evaluate the long-term sustainability and effectiveness of training programs [38]. Incorporating adult learning principles, simulation-based training, train-the trainer models, and interprofessional education may enhance engagement and retention, leading to more meaningful behavior change [39–42]. Standardizing methodologies for assessing training effectiveness will help ensure that educational interventions not only meet competency benchmarks but also lead to measurable improvements in survivor-centered care [17]. 

By continuing to refine human trafficking training for healthcare professionals and evaluating its real-world impact, we can work toward a healthcare system that is better equipped to support survivors and prevent further harm.

### Limitations

There are several limitations that are worth noting when considering the implications of this analysis. First, there were a relatively small number of reviews and trainings that were used to analyze interrater reliability. At the time of the data collection and procedure, this sample of trainings reflected the bulk of readily accessible human trafficking curricula for health care providers. Since that time, there has been a rapid growth in the number of trainings available to clinicians [43]. Some items could be modified to improve clarity and reduce subjectivity of their scores. Furthermore, some of the variability may have been because of the variety of professional roles of the reviewers, including some in roles as clinical trainees.

## Conclusions

Because of the great likelihood that trafficked persons will require health services both while they are in a trafficking situation and once they have exited, there is every reason to invest in training of health care providers as a means to improve the well-being and safety of trafficked persons and related populations. When healthcare workers are not properly trained to recognize trafficking, the crime and its health consequences go unnoticed.

In conclusion, the HT-CAT is a reliable tool for assessing introductory-level human trafficking education of healthcare professionals. HT-CAT can aid credentialing bodies, as well as facilitate implementation of new policies, such as in states that have mandated human trafficking education for health care professionals. HT-CAT can also serve as a valuable resource for medical educators in designing and refining human trafficking curricula, ensuring alignment with best practices. Rigorous assessment of key components of human trafficking trainings for healthcare professionals can help ensure that high quality, relevant education is offered to this group of direct service providers that play a crucial role in identifying and supporting survivors of human trafficking.

## Electronic supplementary material

Below is the link to the electronic supplementary material.

Supplementary Material 1

Supplementary Material 2

## Data Availability

The datasets used during the current study are available from the corresponding author on reasonable request.

## Abbreviations

HT-CAT: Human Trafficking Curriculum Assessment Tool
ICC: Intraclass correlation
U.S.: United States
