# Supplementary material for: A reliability analysis: human trafficking curriculum assessment tool (HT-CAT) for health care provider human trafficking trainings
Source: BMC Med Educ. 2025 Mar 21;25:413. doi: 10.1186/s12909-025-06932-2 (PMC11927203; doi:10.1186/s12909-025-06932-2)
Supplement: Supplementary file 1 — Supplementary Material 1 [file 12909_2025_6932_MOESM1_ESM.docx]

**Additional File A. HT-CAT Instrument - Domains and Items**

| **Full Item Wording** | **Domain** |
| --- | --- |
| Does the training describe the TVPA? | Overview |
| Does the training describe the concepts of force, fraud, or coercion within the context of human trafficking? |  |
| Are all major forms of trafficking identified and discussed? |  |
| Does the training clarify that trafficking does not require crossing international or state borders? |  |
| Does the training differentiate between human trafficking and smuggling? |  |
| Does the training differentiate between human trafficking and consensual commercial sex? |  |
| Does the training include a discussion of the limitations of data on human trafficking? |  |
| Does the training include a discussion on vulnerabilities to trafficking? |  |
| Does the training include a variety of trafficker profiles and their recruitment techniques? |  |
| Does the training describe acute injuries? | Health Impact |
| Does the training describe chronic medical problems? |  |
| Does the training describe mental health issues? |  |
| Does the training describe reproductive and sexual health concerns? |  |
| Does the training describe the impact on quality of life, autonomy, and independence? |  |
| Does the training describe clinical settings in which trafficked persons may be encountered? | Identification and Assessment |
| Does the training describe the challenges and opportunities when interacting with trafficked persons? |  |
| Does the training describe survivor barriers to disclosure? |  |
| Does the training describe provider challenges to identification and response? |  |
| Does the training describe potential red flags of trafficking in persons? |  |
| Does the training describe the role of trauma-informed care in trust-building and communication? |  |
| Does the training discuss measures to keep oneself and patients safe? |  |
| Does the training provide strategies to have private conversations with potential trafficked persons? |  |
| Does the training provide samples of appropriate language to assist with identification? |  |
| Does the training describe the importance of appropriate documentation? |  |

**Additional File A. HT-CAT Instrument - Domains and Items (Continued)**

| **Full Item Wording** | **Domain** |
| --- | --- |
| Does the training describe the importance of healthcare provider role in intervention and response? | Response and Follow-up |
| Does the training describe the importance of survivor-centered, multidisciplinary referrals within the health care organization and with community partners? |  |
| Does the training include a discussion of mandated reporter obligations? |  |
| Does the training include a discussion of the implications of law enforcement involvement? |  |
| Does the training provide information on how to contact your community, local, and/or state resources? |  |
| Does the training include a discussion of the importance of organizational protocols? |  |
| Does the training provide the National Trafficking Hotline number and text number along with any local hotlines? |  |
| Does the training include information on the resources available through HEAL Trafficking? |  |
| Does the training exclude sensationalized imagery? | Design |
| Was the training designed with human trafficking survivor consultation? |  |
