# Supplementary material for: A reliability analysis: human trafficking curriculum assessment tool (HT-CAT) for health care provider human trafficking trainings
Source: BMC Med Educ. 2025 Mar 21;25:413. doi: 10.1186/s12909-025-06932-2 (PMC11927203; doi:10.1186/s12909-025-06932-2)
Supplement: Supplementary file 2 — Supplementary Material 2 [file 12909_2025_6932_MOESM2_ESM.docx]

**Additional File B: Training Name and Website**

| **Training Name** | **Host Organization** |
| --- | --- |
| Emerging Issues on Human Trafficking Webinar: The Public Health Framework | U.S. Administration for Children & Families |
| Rescue and Restore Campaign |  |
| SOAR for Behavioral Health |  |
| SOAR for Health Care |  |
| SOAR for Public Health |  |
| SOAR To Health and Wellness Online Training |  |
| Human Trafficking 101: Dispelling the Myth | American Hospital Association |
| Recognizing and Responding to Human Trafficking in a Health Care Context | Bridging Refugee Youth and Children’s Services |
| Human Trafficking and the Role of the Health Provider | Catholic Health Initiatives |
| Human Trafficking Response Program | CommonSpirit Health Philanthropy |
| Addressing Human Trafficking in Health Care Settings | Essential Access Health |
| An Introduction to Labor and Sex Trafficking: A Health Care and Human Rights Challenge | Futures without Violence |
| Identifying and Supporting Victims of Human Trafficking: An Interactive Resource | Health Education England |
| Human Trafficking: Information and Resources for Emergency Health Care Providers | Humantraffickinged |
| Human Trafficking: The Role of the Medical Professional | Kafayat Adegbenro |
| Human Trafficking 101 | National Association of Pediatric Nurse Practitioners |
| Human Trafficking: The Role of the Health Care Provider | National Health Collaborative on Violence and Abuse |
| Framework for a Human Trafficking Protocol in Health Care Settings | National Human Trafficking Hotline |
| CME on Human Trafficking and Exploitation | NetCE |
| Partners Against Trafficking Humans Videos | Partners Against Trafficking Humans |
| Family Planning: Human Trafficking in the Family Planning Setting | Reproductive Health National Training Center |
| Sex Trafficking in the U.S.: Young Lives, Insane Profit, Yolanda Schlabach, RN | TEDxWillmington |
| Intervention in Human Trafficking in Health Care, Susie Baldwin, MD, MPH | TEDMED |
| Human Trafficking: Identifying and Responding to Victims in the Health Care Setting | U.S. Office of Juvenile Justice and Delinquency Prevention |
